# Plasma microvesicle analysis identifies microRNA 129-5p as a biomarker of heart failure in univentricular heart disease

**DOI:** 10.1371/journal.pone.0183624

**Published:** 2017-08-31

**Authors:** Sweta Ramachandran, Alexander Lowenthal, Carissa Ritner, Shiri Lowenthal, Harold S. Bernstein

**Affiliations:** 1 Department of Pediatrics and Cardiovascular Research Institute, University of California San Francisco, San Francisco, CA, United States of America; 2 Department of Pediatrics and the Mindich Child Health and Development Institute, Icahn School of Medicine at Mount Sinai, New York, NY, United States of America; University of Cincinnati College of Medicine, UNITED STATES

## Abstract

Biomarkers of heart failure in adults have been extensively studied. However, biomarkers to monitor the progression of heart failure in children with univentricular physiology are less well understood. We proposed that as mediators of diverse pathophysiology, miRNAs contained within circulating microvesicles could serve as biomarkers for the presence and progression of heart failure in univentricular patients. To test this, we studied the association of heart failure with elevations in specific miRNAs isolated from circulating microvesicles in a cohort of children with univentricular heart disease and heart failure. We conducted a single site cross-sectional observational study of 71 children aged 1 month-7 years with univentricular heart disease and heart failure. We demonstrated that levels of miR129-5p isolated from plasma microvesicles were inversely related to the degree of clinical heart failure as assessed by Ross score. We then showed that miR129-5p levels are downregulated in HL1 cells and human embryonic stem cell-derived cardiomyocytes exposed to oxidative stress. We demonstrated that bone morphogenetic protein receptor 2, which has been implicated in the development of pulmonary vascular disease, is a target of miR129-5p, and conversely regulated in response to oxidative stress in cell culture. Levels of miR129-5p were inversely related to the degree of clinical heart failure in patients with univentricular heart disease. This study demonstrates that miR129-5p is a sensitive and specific biomarker for heart failure in univentricular heart disease independent of ventricular morphology or stage of palliation. Further study is warranted to understand the targets affected by miR129-5p with the development of heart failure in patients with univentricular physiology.

## Introduction

Biomarkers of heart failure (HF) resulting from ischemic cardiomyopathy in adults have been extensively studied. Biomarkers to monitor the progression of HF in infants and young children with univentricular physiology, however, are less well understood. This may be due to the etiology of HF in this population, specifically myocardial dysfunction resulting from the altered hemodynamics of the pulmonary and systemic circulations [1] and pulmonary vascular disease [2].

Small, noncoding microRNAs (miRNAs) are important posttranscriptional regulators of gene expression, with each miRNA predicted to regulate hundreds of mRNA target genes [3,4]. As such, miRNAs are known to influence biological and metabolic processes that are dysregulated in various diseases [5]. However, the ability to evaluate circulating miRNA levels in patients is made difficult by their relatively low concentration in a large volume of circulating plasma.

Microvesicles that directly bud from the plasma membrane, and exosomes released via exocytosis from multivesicular bodies of the endosome, play key roles in intercellular communication [6]. These small vesicles are known to carry diverse proteins as well as nucleic acids such as miRNAs, and circulating levels of microvesicles are elevated in various disorders, including cardiovascular and metabolic disease [7]. As such, both the vesicles themselves as well as their contents have been deemed potentially useful as prognostic and diagnostic biomarkers for various diseases.

We proposed that as mediators of diverse pathophysiology, miRNAs contained within microvesicles could serve as biomarkers for the presence and progression of HF in univentricular patients. To test this hypothesis, we measured the association of HF with elevations in specific miRNAs isolated from circulating microvesicles in a cohort of children with univentricular heart disease and HF.

## Methods

### Clinical study design

A cross-sectional observational study utilizing a secondary study base was conducted at a single site as previously described [8]. Briefly, all children age 1 month-7 years with univentricular congenital heart disease presenting to the University of California San Francisco (UCSF) between February 2007 and December 2010 were eligible for the study. This study was approved by the UCSF Institutional Review Board, and written consent was obtained from guardians of all subjects.

Each child was assigned a Ross score [9–11] to determine the presence of clinical HF immediately prior to phlebotomy (Table 1). As previously discussed [8], HF scoring was recorded independent of presumed mechanism, since the goal was to evaluate biomarkers in univentricular patients that would be clinically useful across etiologies. Predictor measurement occurred subsequent to outcome determination, allowing assessors to be blinded to plasma miRNA levels. The primary outcome was HF (Ross score ≥3) versus no HF (Ross score ≤2). The relationship between raw Ross score and miRNA expression level was summarized using scatter plots.

**Table 1:** Patient data.

Ventricular Morphology	Stage of Palliation*	Total Patients	Female/ Male	Median Age, months (Range)	Ross Score <3^†^	Ross Score 3–9^‡^
**Right**	*Total*	51	22/29	16 (2–80)	36	15
	Stage I	19	9/10	4.4 (2–8)	13	6
	Stage II	24	11/13	35 (5–72)	17	7
	Stage III	8	2/6	66 (34–80)	6	2
**Left**	*Total*	18	4/14	44 (3–78)	11	7
	Stage I	4	1/3	5 (3–11)	4	0
	Stage II	11	4/7	45 (17–69)	5	6
	Stage III	3	0/3	73 (69–78)	2	1
**Indeterminate**	*Total*	2	1/1	60 (56–66)	2	0
	Stage I	0	0/0	0	0	0
	Stage II	2	1/1	60 (56–66)	2	0
	Stage III	0	0/0	0	0	0

* Stage I: stabilization of aortic and pulmonary blood flow, *e*.*g*., Norwood, Sano, pulmonary artery band, Blalock-Taussig shunt, central shunt; Stage II: establishment of partial cavopulmonary circulation between the superior vena cava and pulmonary arteries, *e*.*g*., Glenn shunt, Kawashima; Stage III: completion of cavopulmonary circulation, *e*.*g*., Fontan.

^†^ Clinical signs of HF absent.

^‡^ Clinical signs of HF present.

### Human embryonic stem cell-derived cardiomyocyte culture

The H9 (WA09; WiCell) human embryonic stem cell (hESC) line was maintained on irradiated mouse embryonic fibroblast feeder cells in medium comprised of Knockout DMEM (Invitrogen) supplemented with 20% Knockout Serum Replacement (Invitrogen), 2 mM L-glutamine, 0.1 mM nonessential amino acids, 0.1 mM β-mercaptoethanol, and 15 ng/mL recombinant human FGF-basic (R&D Systems), according to published methods [12]. Differentiation was initiated by human embryoid body (hEB) formation in suspension as previously described [13]. Briefly, colonies of hESCs were dissociated into clusters by exposure to Collagenase IV (Sigma-Aldrich) and then allowed to differentiate in a medium comprised of Knockout DMEM (Invitrogen) supplemented with 20% Defined Fetal Bovine Serum (Hyclone), 2 mM glutamine, 0.1 mM non-essential amino acids, and 0.1 mM β-mercaptoethanol. After 4 days in suspension, hEBs were attached to gelatin-coated 12-well culture plates and allowed to differentiate for an additional 17 days [14]. On day 21, beating foci representing hESC-derived cardiomyocytes were isolated and plated on gelatin-coated plates for 24 hours before treatment and collection for RNA isolation.

### HL1 cell culture

HL1 cells were maintained according to published methods [15]. Briefly, cells were plated in T25 flasks coated with 25μg fibronectin (Sigma-Aldrich, F1141) in 2 mL 0.02% bovine gelatin (Sigma-Aldrich, G9391) in water. Cells were maintained in Claycomb Medium (Sigma-Aldrich, 51800C) supplemented with 10% FBS (Sigma-Aldrich, F2442, Batch 11A568, US origin), 100 μg/mL Penicillin-Streptomycin (Sigma-Aldrich, P4333), 0.1mM norepinephrine (Sigma-Aldrich, A0937), and 2mM L-glutamine (Sigma-Aldrich, G7513). Norepinephrine stock (10mM) was made up in 30mM ascorbic acid (Sigma-Aldrich, A7506) and filtered using a 0.2um syringe filter (Gelman Sciences, 4192). Cells were grown at 37°C in 5% CO2 and were passaged upon reaching confluence every 2–3 days by dissociating into single cells using 0.05% Trypsin in 0.02% EDTA-Na (Sigma-Aldrich, T3924).

### Cell culture hypoxia model

HL1 or hESC-derived cells were subjected to hypoxic conditions by culture in a hypoxia chamber (BioSpherix Xvivo System, model G300C) in 1% O2/5% CO2 at 37°C for 8, 16, or 24 hrs [16]. Alternatively, H_2_O_2_ (Sigma-Aldrich) was added to supplemented Claycomb growth medium for 3 hrs at a final concentration of 100μM [17].

### Trypan blue exclusion assay

To determine whether prolonged periods of hypoxia affected cell viability, a trypan blue exclusion test was performed. An aliquot of the cell suspension was mixed with an equal volume of 0.4% trypan blue (Sigma-Aldrich, T8154), and after a 3 min incubation, unstained (viable) and stained (nonviable) cells were counted using a hemacytometer to determine the percentage of viable cells.

### RNA isolation from plasma exosomes

miRNA expression was assessed from plasma combined from multiple patients based on Ross score at time of collection, or individual patient plasma. Briefly, plasma samples were centrifuged at 3000xg to precipitate cell debris. Supernatants were pipetted to fresh, sterile microfuge tubes. For pooled plasma analysis, samples from 4–6 patients with identical Ross scores were combined into a single sample of 200ul total volume in a fresh microfuge tube. For example, a panel of five patient samples would be collected using 40ul of each sample. For individual patient samples, a total volume of 100ul plasma was transferred to a fresh tube.

A 25.2ul volume of ExoQuick Exosome Precipitation reagent (System Biosciences, EXOQ20A-1) was added to each tube per 100ul plasma to be analyzed, then suspended by inverting 4–6 times. The mixture was incubated at 4°C for 12h, then centrifuged at 1500xg for 30 min to pellet exosomes. The supernatant was removed by aspiration, taking care not to disturb the pellet. The exosome pellet was resuspended in 20ul nuclease-free water per 100ul plasma and stored in 10ul aliquots before proceeding to miRNA isolation.

Following exosome isolation, miRNA was isolated from exosomes using SeraMir RNA isolation kit (System Biosciences, RA800A-1). Proceeding with one exosome aliquot, the sample was centrifuged at 1500xg for 10 min to repellet exosomes and supernatant removed. A volume of 100ul lysis buffer was added to the pellet then vortexed for 5 sec. Following a 5 min incubation at room temperature, 40ul 100% ethanol was added to the mixture and vortexed for 10 sec. The total volume was transferred to a preassembled spin column and centrifuged at 7000xg for 1min. The column was washed twice with 80ul wash buffer and centrifuged at 700xg for 1 min for each wash. The spin column was then transferred to a fresh RNAse-free collection tube. RNA was eluted with 20ul Elution Buffer according to the manufacturer’s instructions. RNA yield was determined using a NanoDrop ND-1000 (Nanodrop Technologies, ND Software version 3.8.1), with unused Elution Buffer as blank control.

### mRNA and miRNA isolation from cell culture

For analysis of mRNA expression, total RNA was isolated from HL1 cells or hESCs using the Taqman Gene Expression Cells-to-CT kit (Ambion). Relative expression was determined using TaqMan Assay (Applied Biosystems) on an ABI 7300 Real-Time PCR system with the following primer pairs (ABI): VEGFA (Hs00900055_ml), ADRB2 (Hs00240532_s1), GLUT1 (Hs00892681_m1), BMPR2 (Hs00176148_ml), and GAPDH (4326317E). Cycle times to detection were normalized against GAPDH and relative changes were calculated using ABI Version 1.4 Sequence Detection Software.

For analysis of miRNA expression, miRNAs were isolated from HL1 cells or hESCs using the mirVana miRNA Isolation kit (Ambion), and cDNA was reverse transcribed using the TaqMan MicroRNA RT kit (ABI). Following linear pre-amplification of miRNA sequences using the Applied Biosystems Preamplification system, relative expression was determined using singleplex TaqMan Assays (ABI) with primer sets for miR-129 (ABI; 000590), miR-18b (ABI; 002310), miR-423-5p (ABI; 002340), miR-622 (ABI; 001553), and miR-452 (ABI; 002330). Cycle times to detection were normalized against two reference sequences, RNU44 (001094) and RNU48 (001006), and relative changes were calculated as described [12].

### Dual-luciferase assay

Luciferase assays were performed in whole HL1 cell lysates using the dual luciferase reporter assay system (Promega) as described previously [14]. pRL-TK (Promega) encoding constitutively expressed Renilla reniformis luciferase was included in each transfection to normalize for transfection efficiency. At the time of miR-129 mirVana miRNA mimic (Ambion, MC10195) transfection (30nM final concentration), HL1 cells were co-transfected with 0.5μg pmiR-129-5p-Luc reporter plasmid (Signosis, binding site sequence: 5’GCAAGCCCAGACCGCAAAAAG3’) together with 50ng of pRL-TK using DharmaFECT Duo (Thermo Scientific, T-2010-02). pmiR-129-5p-Luc expresses Photinus pyralis luciferase under control of a CMV promoter, regulated by the presence of a miR-129 binding site within the 3’ UTR of the luciferase coding sequence. To assess the specificity of the miRNA-binding site of the pmiR-129-5p-Luc reporter plasmid, a custom luciferase reporter vector with a scrambled binding site (Signosis, scrambled binding site sequence: 5’CAGACGAACCGGACAACGAAC3’) was transfected into HL1 cells along with the miR-129 miRNA mimic and pRL-TK.

After 48 hrs, the cells were collected and cell lysates were assayed for Photinus and Renilla luciferase activities using a Monolight 2010 luminometer (Analytical Luminescence Laboratory). Photinus luciferase activity was normalized against Renilla luciferase activity and expressed as relative light units. Assays were performed in triplicate and repeated at least three times.

### Statistical analysis

For comparison of quantitative real-time PCR and relative luciferase activity, analysis of variance (ANOVA) with Fisher's post-hoc test was used. Where ANOVA indicated significant differences among groups, multiple comparisons were made using Student's t- test with Bonferroni correction. A p-value less than 0.05 was considered significant. A receiver operating characteristic (ROC) curve was generated to evaluate miR129-5p as a biomarker in the entire cohort. The assay was deemed useful if it met a pre-specified threshold for c-statistic of 0.75 (graph area under the curve). All statistical analyses were performed using Stata 10 (StataCorp LP, College Station, TX).

## Results

We approached 91 patients with univentricular disease meeting inclusion criteria and presenting to UCSF between February 2007 and December 2010 for enrollment. Details of the 71 children studied have been described previously [8]. Patients were assigned Ross scores as a measure of clinical HF as previously described [8] and the surgical stage of palliation was noted (**Table 1**). Of the 49 children free of clinical HF, the median Ross score was 1±0.8. For the 22 children with clinical HF, the median Ross score was 4±1.7. The overall prevalence of HF in the included sample was 31%, with morphology-specific prevalence of 30% in those with single RV and 39% in those with single LV.

Early attempts to assay for miRNA expression in the circulation have had variable success because of their dilution in total blood. Since microvesicles have been shown to concentrate small nucleic acids circulating in bodily fluids, and provide access to these for quantitative assessment, we isolated microvesicles from the sera of univentricular patients to assess the concentrations of circulating, cardiac-specific miRNAs. Subsequent NanoSight analysis of microvesicles in the 50-250nm size range ultimately proved informative (**S1 Fig**). Size was established by comparison to particle size standards using nanoparticle tracking analysis ([18]; **S1 Video** and **S2 Video**).

**Fig 1:**
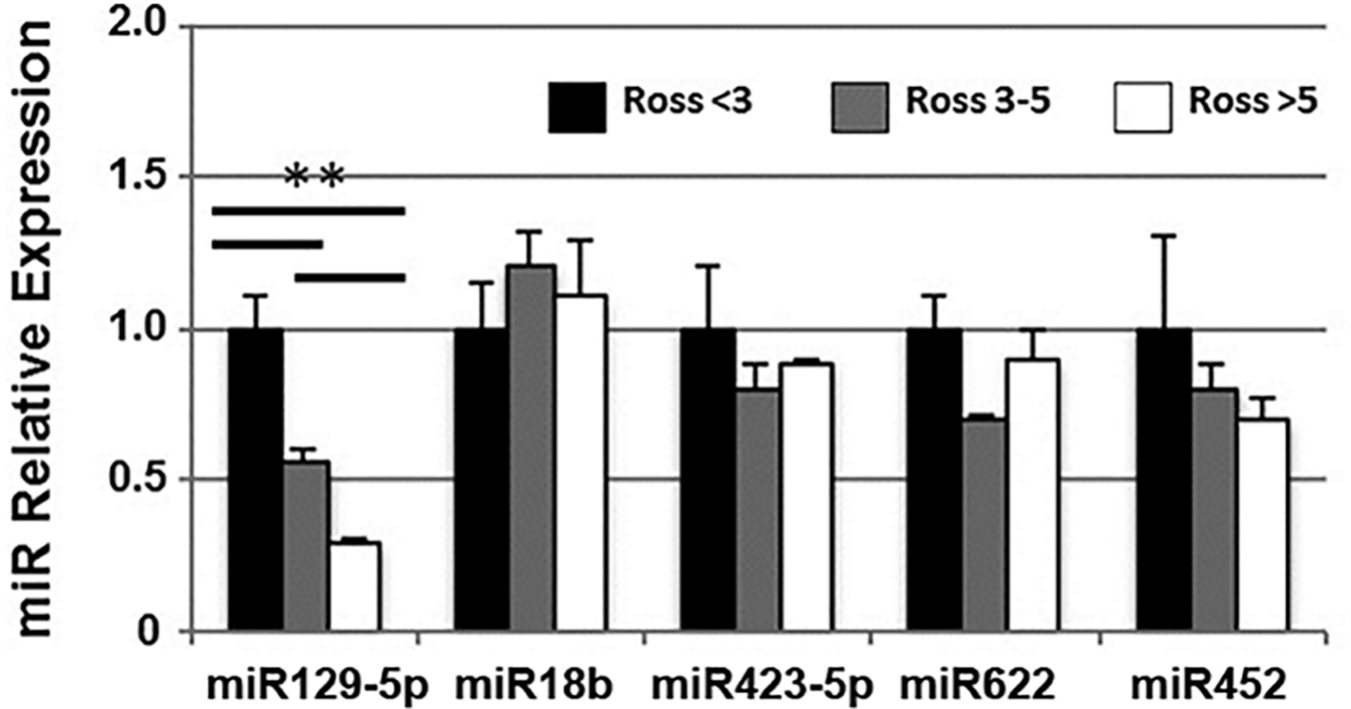
miRNA expression in pooled samples from univentricular heart failure patients. Pooled sera from patients with Ross scores <3, 3–5, and >5 were evaluated for expression of miRNAs previously implicated as biomarkers of HF in adult patients. miR129-5p showed a decrease in expression with increasing Ross score. Data shown are mean ± SEM (n = 4–6); **p<0.01.

**Fig 2:**
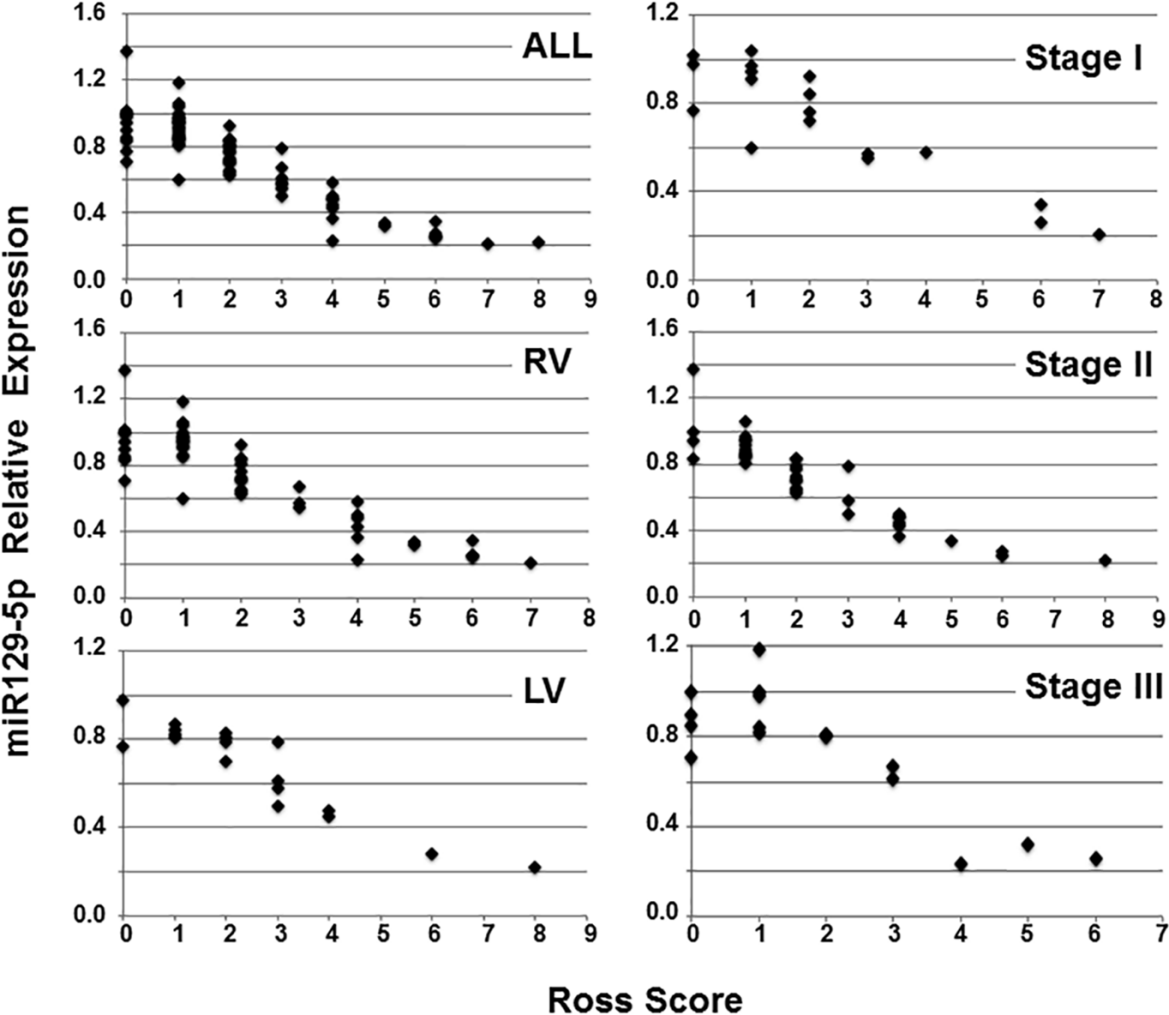
miR129-5p expression by ventricular morphology and stage of palliation. Sera from individual patients were evaluated for miR129-5p expression and plotted relative to Ross score. Individual plots of patients stratified by ventricular morphology (ALL, all patients; RV, right ventricular morphology; LV, left ventricular morphology) and stage of surgical palliation (Stage I, stabilization of aortic and pulmonary blood flow; Stage II, establishment of partial cavopulmonary circulation between the superior vena cava and pulmonary arteries; Stage III, completion of cavopulmonary circulation) are shown. miR129-5p expression was downregulated with increasing Ross score independent of ventricular morphology or stage of palliation. Mean data are shown (n = 3).

**Fig 3:**
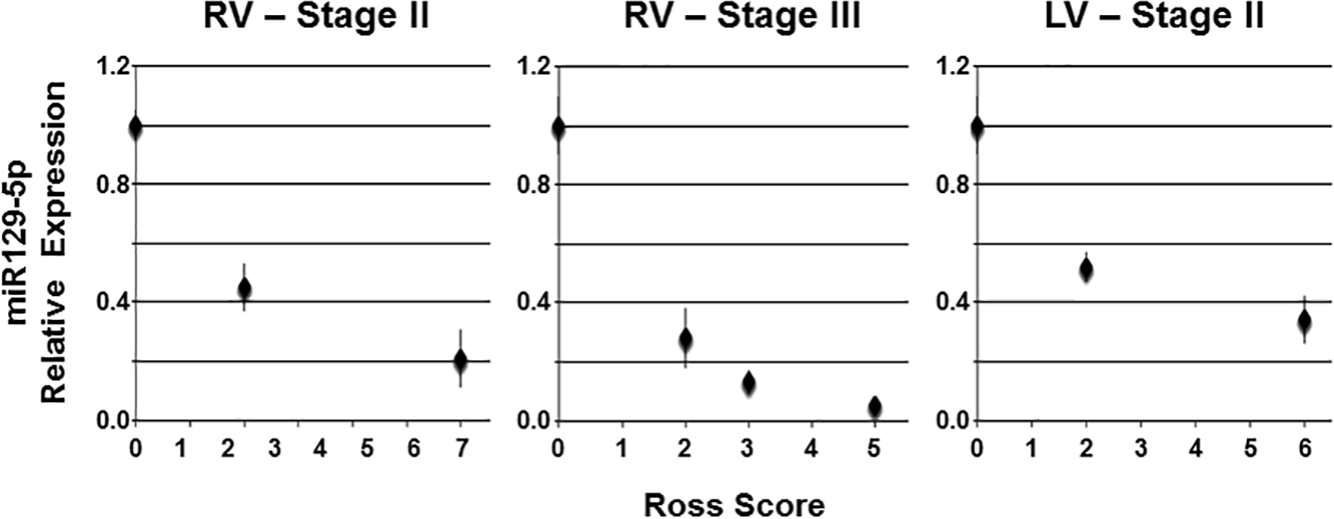
Serial miR129-5p expression in univentricular heart failure patients. Serial plasma samples from three patients were evaluated for miR129-5p expression and plotted relative to Ross score at the time of sample collection. Two patients had undergone partial cavopulmonary connection surgery (Stage II), one with morphological RV and one with morphological LV. One patient had undergone completion of cavopulmonary connection (i.e., extracardiac Fontan; Stage III) and had a morphological RV. In each patient, miR129-5p expression was downregulated with increasing Ross score. Data shown are mean ± SEM (n = 3).

Aliquots of sera were pooled from patients with Ross scores <3, 3–5, and >5 for expression analysis of miR129-5p, miR18b, miR423-5p, miR622, and miR452. These miRNAs were chosen based on previous reports that they may serve as biomarkers for symptomatic heart failure in adults [19]. A dose-dependent inverse relationship was observed between Ross score and miR129-5p within this cohort (**Fig 1**). To further define the relationship between miR129-5p expression and clinical heart failure, individual patient sera were analyzed for miR129-5p expression and stratified by type of single ventricle and stage of surgical palliation (**Fig 2**). This demonstrated that miR129-5p levels declined with increasing Ross score independent of ventricular morphology or stage of palliation. ROC curve analysis of miR129-5p data for the entire cohort exceeded our pre-specified threshold of ≥75% area contained by the curve (c-statistic 98%, P<0.0001; **S2 Fig**). A decrease in relative expression of miR129-5p to ≤0.58 predicts the presence of clinical HF with 85% sensitivity and 100% specificity. In addition, samples were obtained from 3 individuals at different times during their disease course at which time a Ross score also was calculated. In each of these individuals, serial measurements showed that miR129-5p level decreased with increasing Ross score (**Fig 3**).

**Fig 4:**
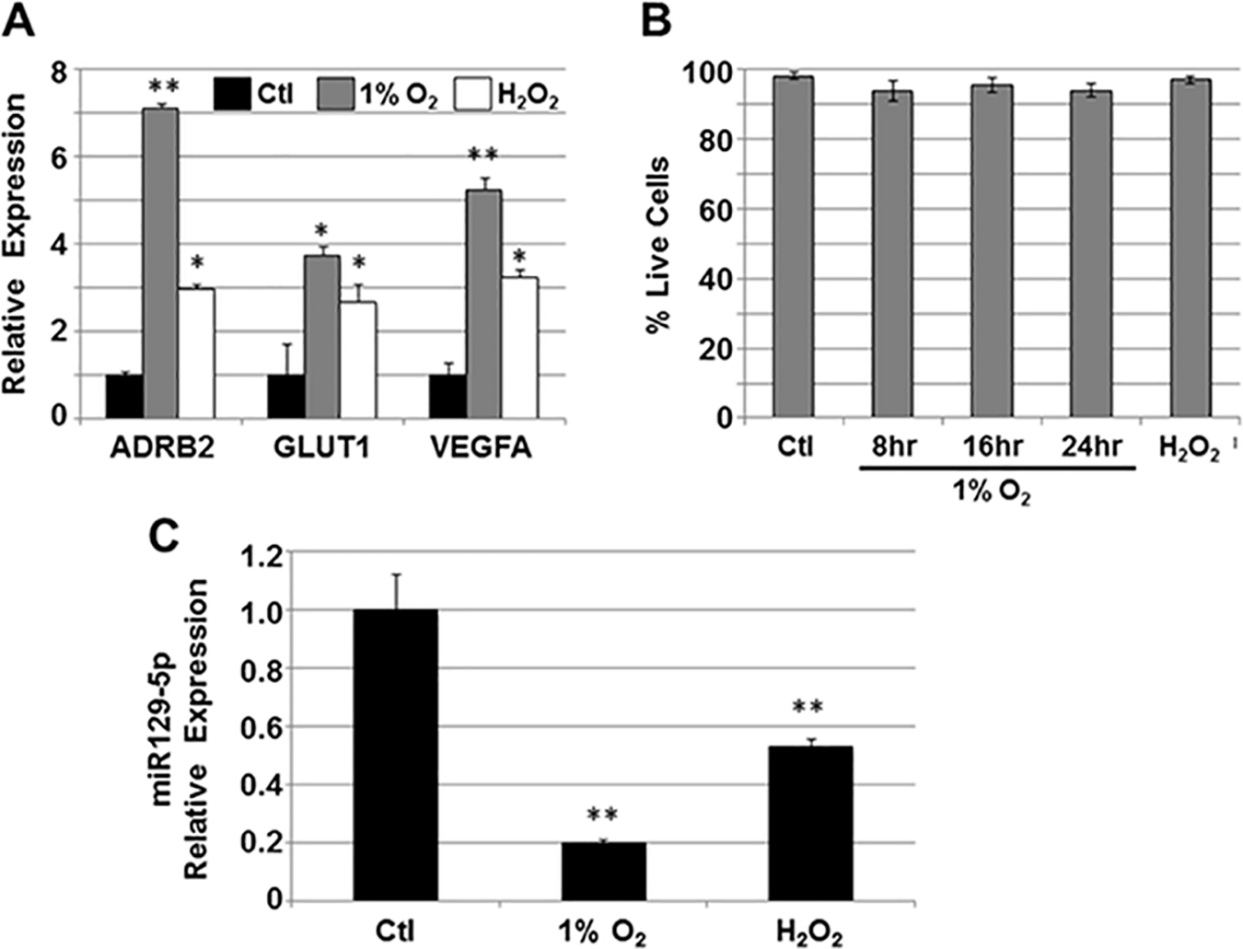
miR129-5p expression in HL1 cells with oxidative stress. A) HL1 cells were grown in 1% O_2_ or 100 μM H_2_O_2_ and relative expression of vascular endothelial growth factor A (VEGFA), glucose transporter 1 (GLUT1), and β2-adrenergic receptor (ADBR2) were assessed by qPCR. An increase in expression of all three hypoxia-mediated genes was seen under both conditions. Data shown are mean±SEM (n = 3); *p<0.05; **p<0.01. B) To demonstrate viability of HL1 cells grown in 1% O_2_ or H_2_O_2_, cells were stained with trypan blue and percent live cells (trypan blue-excluding) were calculated. Neither condition significantly affected HL1 cell viability. Data shown are mean±SEM (n = 3). C) miR129-5p expression in HL1 cells grown in 1% O_2_ or H_2_O_2_ was analyzed by qPCR. Under both conditions, miR129-5p expression was downregulated. Data shown are mean±SEM (n = 3); **p<0.01.

To evaluate whether changes in miR129-5p expression in response to HF may originate from the myocardium, HL1 cells representing human adult cardiomyocytes [15] were grown under hypoxic conditions (1% O_2_) or subjected to oxidative stress (100 μM H_2_O_2_) (**Fig 4**). Under both conditions, HL1 cells expressed higher levels of β2-adrenergic receptor (ADRB2), glucose transporter 1 (GLUT1), and vascular endothelial growth factor A (VEGFA; **Fig 4A**), all known to be upregulated in cardiomyocytes exposed to hypoxia *in vitro* [20], and in response to failing myocardium *in vivo* [21]. Similarly, miR129-5p expression decreased in HL1 cells under both conditions (**Fig 4C**).

**Fig 5:**
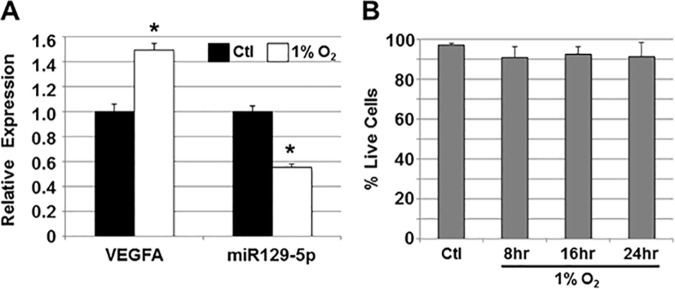
miR129-5p expression in hESC-derived cardiomyocytes with hypoxia. A) hESC-derived cardiomyocytes were cultured in 1% O_2_ and relative expression of vascular endothelial growth factor A (VEGFA) and miR129-5p were assessed by qPCR. Upregulation of hypoxia-mediated VEGFA and downregulation of miR129-5p was seen. Data shown are mean±SEM (n = 3); *p<0.05. B) To demonstrate viability of hESC-derived cardiomyocytes grown in 1% O2, cells were stained with trypan blue and percent live cells (trypan blue-excluding) were calculated. Hypoxia did not significantly affect cell viability. Data shown are mean±SEM (n = 3).

To extend these findings, we also exposed cardiomyocytes derived from hESCs to 1% O_2_, and similarly observed an increase in expression of VEGFA, consistent with an hypoxic response, as well as a decrease in miR129-5p expression (**Fig 5**).

**Fig 6:**
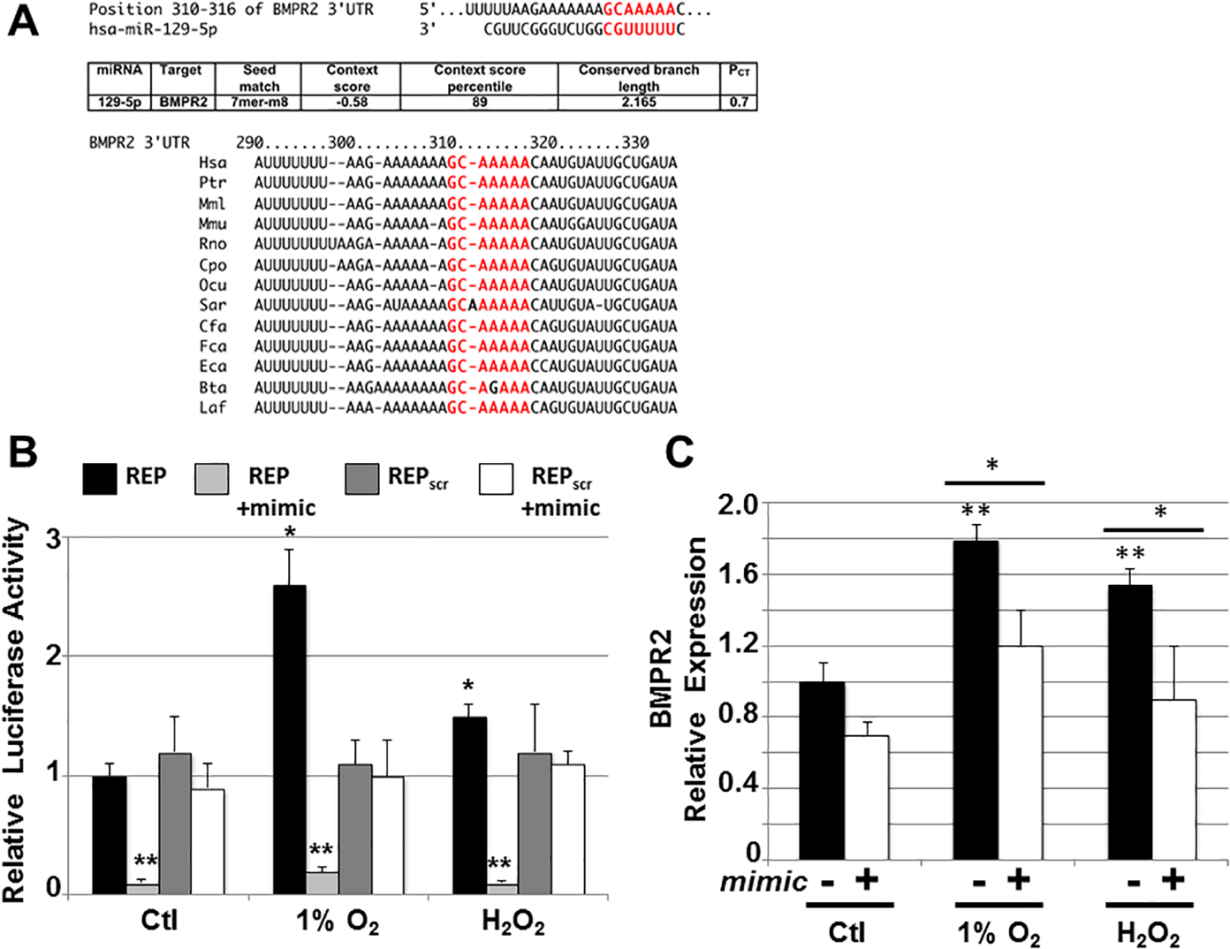
Bone morphogenetic protein receptor 2 is a target of miR129-5p in HL1 cells. A) *In silico* analysis of potential miR129-5p targets using Target Scan Human identified bone morphogenetic protein receptor 2 (BMPR2) as a potential target. Conservation among mammals of the miR129-5p binding site in the 3’UTR of BMPR2 is shown below. Bta, cow; Cfa, dog; Cpo, guinea pig; Eca, horse; Fca, cat; Hsa, human; Laf, elephant; Mml, rhesus; Mmu, mouse; Ocu, rabbit; Ptr, chimpanzee; Rno, rat; Sar, shrew. B) HL1 cells transfected with BMPR2 3’UTR reporter (REP) show increased luciferase activity in 1% O_2_ and 100 μM H_2_O_2_. Co-transfection with miR129-5p (mimic) downregulates luciferase activity. Reporter plasmid expressing luciferase with scrambled BMPR2 3’UTR sequence (REP_scr_) was used as control. Data shown are mean±SEM (n = 3); *p < 0.05; **p<0.01. C) HL1 cells were grown in 1% O_2_ or 100 μM H_2_O_2_ and relative expression of BMPR2 in the presence and absence of transfected miR129-5p (mimic) was assessed by qPCR. An increase in BMPR2 expression was seen under both conditions of oxidative stress, and this increase was suppressed by overexpression of miR129-5p. Data shown are mean±SEM (n = 3); *p<0.05; **p<0.01.

miRNAs are known to bind to specific sites within the 3’ untranslated region (3’UTR) of a target gene and downregulate transcription of the target [4]. TargetScan (version 6.2) was used to search the 3’UTRs of expressed genes for the presence of 7/8-mer sites that matched the seed region of miR129-5p. This identified an evolutionarily conserved sequence in the 3’ UTR of the gene encoding bone morphogenetic protein receptor 2 (BMPR2; **Fig 6A**), a serine/threonine receptor kinase for which inactivating mutations have been linked to pulmonary hypertension [22]. Using a dual-luciferase reporter assay in HL1 cells, we demonstrated that transcriptional activity under control of the BMPR2 3’UTR was upregulated under hypoxic or oxidative stress conditions, however, this response was inhibited with overexpression of miR129-5p (**Fig 6B**). We also showed that overexpression of miR129-5p results in downregulation of BMPR2 transcript expression in HL1 cells cultured under these conditions (**Fig 6C**).

## Discussion

This study demonstrated that microvesicles can be interrogated as a source of miRNAs released into the circulation with HF, that there is an inverse relationship between levels of miR129-5p in circulating microvesicles and the degree of HF in pediatric patients with univentricular heart disease, that miR129-5p is similarly downregulated in cultured human cardiomyocytes and cardiomyocytes derived from hESCs exposed to oxidative stress, and that BMPR2 expression is likely regulated by miR129-5p in the setting of cardiomyocyte stress.

Microvesicles, which are released by most cells, play an important role as intercellular messengers by transferring nucleic acids, including miRNAs, and proteins between cells [23]. Beyond their role in delivering molecules between cells, their contents have more recently been explored as a source of potential biomarkers for the presence and progression of various disease states [24]. Given the dilution of candidate biomarker miRNAs in the circulation, and the limits of sample collection in pediatric patients, we sought to demonstrate that by first isolating microvesicles from plasma samples, we could enrich for the targets of our analysis. This allowed us to evaluate several candidate miRNAs previously associated with HF in adult patients, and specifically identify miR129-5p as an informative biomarker for HF in children with univentricular heart disease.

In previous studies of potential biomarkers for HF in this population, we found that both the morphology of the single ventricle and the stage of palliation affected the sensitivity of the biomarker for detecting HF. For example, high-sensitivity C-reactive protein (hsCRP) was sensitive (100%) and specific (63%) for detecting HF in univentricular patients with a morphological LV, but not a morphological RV [25]. In addition, hsCRP was sufficiently sensitive for detecting HF in univentricular patients at stage II of palliation (sensitivity 100%, specificity 57%), but less so at other stages of palliation [25]. Similarly, we determined that B-type natriuretic peptide (BNP) and N-terminal pro-BNP were reliable tests for clinical HF in children with univentricular congenital heart disease of RV morphology regardless of stage of palliation [8]. In contrast, the current study demonstrates that miR129-5p levels vary with degree of clinical HF in univentricular heart disease independent of ventricular morphology and stage of palliation. This suggests that it potentially can be used as a sensitive (85%) and specific (100%) circulating biomarker in a broader population of patients with univentricular disease.

Our study was limited by our clinical assessment tool, i.e., the Ross score has not been specifically validated in the univentricular population. However, a generalized HF grading system based on the specific criteria of the Ross score, the Ross classification of HF, has been used in several previous studies of HF, including recent multicenter trials of carvedilol and enalapril therapy for pediatric HF that included univentricular patients [26,27]. As such, we considered the combined criteria included in the Ross score the most relevant outcome measure for this study.

BMPR2 is known to play a role in pulmonary vascular remodeling in pulmonary hypertension [28,29]. Genetic studies have shown that inactivating BMPR2 mutations occur in the majority of patients with familial pulmonary hypertension and up to 25% of patients with idiopathic pulmonary hypertension [28,29]. Preclinical and clinical studies of univentricular physiology have shown that the aberrant circulation in these patients lead to pulmonary vascular disease, and that this in turn may contribute to the development of ventricular dysfunction and failure [2]. While we see a decrease in miR129-5p levels with worsening HF symptoms, this should result in disinhibition of BMPR2 expression contrary to what occurs with inactivating mutations leading to idiopathic pulmonary hypertension. Based on our observations, therefore, we speculate that the mechanism by which pulmonary hypertension develops in patients with univentricular heart disease may be distinct from idiopathic pulmonary vascular disease. However, further interrogation of the pathway in relevant models would be needed to test this hypothesis.

In conclusion, while biomarkers are used to diagnose disease, stratify patient risk, and measure response to therapy, newly identified biomarkers may also provide mechanistic insight into disease processes. In this case, miR129-5p appears to serve as a potential biomarker for HF in univentricular heart disease, and further study of miR129-5p targets may elucidate the mechanism by which patients with univentricular physiology develop HF and its pulmonary vascular sequelae.

## Supporting information

S1 Fig Size distribution of microvesicles isolated from patient plasma. A microvesicle pellet isolated from 100μl plasma resuspended in 20μl nuclease-free water was analyzed by nanoparticle tracking using a NanoSight LM10 (NanoSight Ltd., Amesbury, UK) according to published methods [18]. A representative profile of particle size and concentration is shown.
(TIF)

S2 Fig ROC analysis of miR129-5p versus Ross scores. False-positive results (1-specificity) were plotted against true-positive results (sensitivity) for 71 patients. Area under the curve (c-statistic) was 0.98, which was highly significant (p<0.0001).
(TIF)

S1 Video Real time nanoparticle tracking analysis of standard particles approximating microvesicles. Standard 100nm polystyrene beads are visualized as single points of light using a NanoSight LM10 to evaluate size and quantity.
(MOV)

S2 Video Real time nanoparticle tracking analysis of microvesicles isolated from patient plasma. A microvesicle pellet isolated from 100μl plasma resuspended in 20μl nuclease-free water was analyzed by nanoparticle tracking using a NanoSight LM10. A representative acquisition is shown.
(MOV)
