# Supplementary figures and images for: Plasma microvesicle analysis identifies microRNA 129-5p as a biomarker of heart failure in univentricular heart disease
Source: PLoS One. 2017 Aug 31;12(8):e0183624. doi: 10.1371/journal.pone.0183624 (PMC5578659; doi:10.1371/journal.pone.0183624)

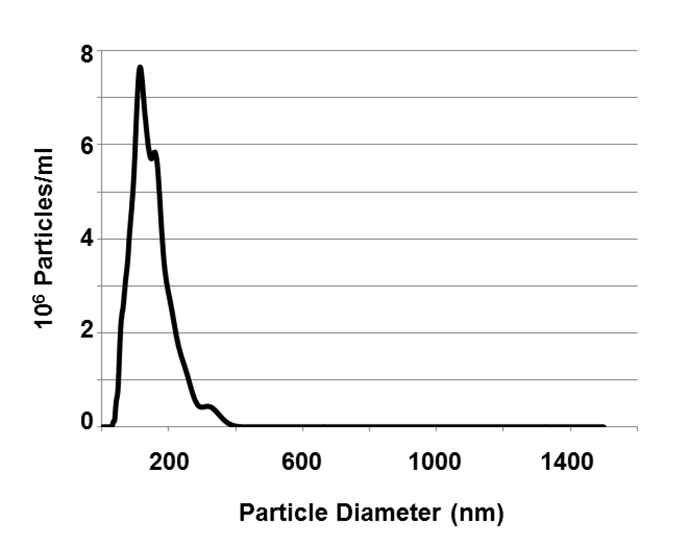

Supplement: S1 Fig — A microvesicle pellet isolated from 100μl plasma resuspended in 20μl nuclease-free water was analyzed by nanoparticle tracking using a NanoSight LM10 (NanoSight Ltd., Amesbury, UK) according to published methods [18]. A representative profile of particle size and concentration is shown. (TIF) [file pone.0183624.s001.TIF]

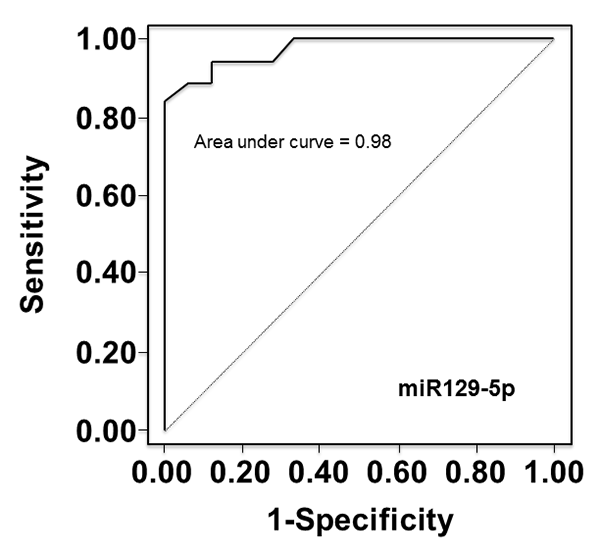

Supplement: S2 Fig — False-positive results (1-specificity) were plotted against true-positive results (sensitivity) for 71 patients. Area under the curve (c-statistic) was 0.98, which was highly significant (p<0.0001). (TIF) [file pone.0183624.s002.TIF]
